# Supplementary material for: HDAC Inhibition Restores Response to HER2-Targeted Therapy in Breast Cancer via PHLDA1 Induction
Source: Int J Mol Sci. 2023 Mar 25;24(7):6228. doi: 10.3390/ijms24076228 (PMC10094256; doi:10.3390/ijms24076228)
Supplement: Supplementary file 1 [file ijms-24-06228-s001.zip › ijms-2296276 - supplementary tables.pdf]

International Journal of Molecular Sciences

MDPI

Supplementary Table S1

Cell Line	Origin	Mutations
HCC1954	Breast ductal carcinoma, *HER2* amplified	$PI3K^{H1047R}$, $P53^{Y163C}$
SKBR3	Breast adenocarcinoma, pleural effusion, *HER2* amplified	$P53^{R175H}$
MFE-296	Endometrial adenocarcinoma	$FGFR2^{N550K,K310R}$, $PTEN^{P130Q, N323fs*}$, $PI3K^{P539R,I20M}$
ANC3A	Endometrial adenocarcinoma, lymph node metastasis	$FGFR2^{N550K,K310R}$, $PTEN^{R130fs*1}$, $p85\alpha^{R557\_D560del}$

Supplementary Table S2

Primer Pair	Forward Primer	Reverse Primer
Control	CCCATGGGAATGCCTTGTCT	TGCCCTTCAATCTTCACGCT
PHLDA1_1	CGTCCCACTTCCTCAAGTCC	TCTCGGAGGGTTCTTAGCCA
PHLDA1_2	TCAAGTGTGCAAAGCTCTCG	GGCCTTTTGACTGATTTGTGAGT
PHLDA1_3	TTGAATGGGCCATCTTCCCC	CCAAGTTAATGAGGCGTGCG
PHLDA1_4	CTCCATACAGGAGCCGAACC	CGGGCAAGACAAGGTAAGGT
PHLDA1_5	CCCGTCTTCGGCTTTCTTCT	AGATCTGGCTAGGCCTTGGA
PHLDA1_6	TCCAACTGCTTACTGCCGAA	AGTGTTCCCTTGCCTGGTATG
PHLDA1_7	CATGATTCCGGTGGCCCTAT	CCCTCTTGGTGGCTGGATTT
