# Supplementary material for: HDAC Inhibition Restores Response to HER2-Targeted Therapy in Breast Cancer via PHLDA1 Induction
Source: Int J Mol Sci. 2023 Mar 25;24(7):6228. doi: 10.3390/ijms24076228 (PMC10094256; doi:10.3390/ijms24076228)
Supplement: Supplementary file 1 [file ijms-24-06228-s001.zip › ijms-2296276_supplementary_figures.pdf]

Supplementary Figure S1

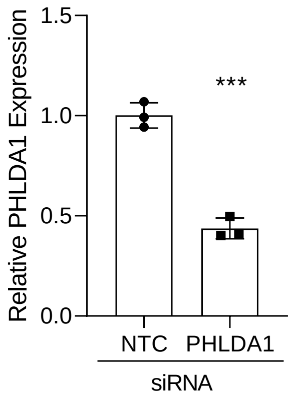

Supplementary Figure S1. PHLDA1 siRNA Validation. QPCR analysis of PHLDA1 mRNA expression in HCC1954 cells transfected with either a non-targeting control (NTC) or PHLDA1 siRNA for 48 hours. Data representative of three biological experiments.

Supplementary Figure S2

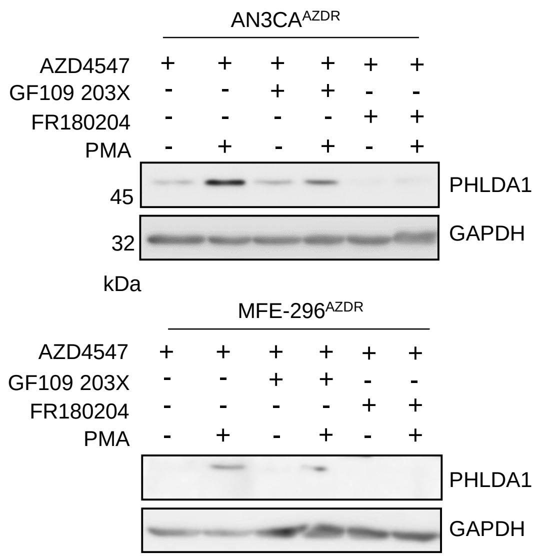

Supplementary Figure S2. Western blot analysis of PHLDA1 expression in AZD4547 resistant AN3CA and MFE-296 cells (AN3CA^{AZDR} and MFE-296^{AZDR}) cultured in 1 µM AZD4547 and treated for 48h with 20 nM PMA in the presence and absence of either 5 µM GF109203X or 40 µM FR180204. Bands are representative of three independent experiments.

Supplementary Figure S3

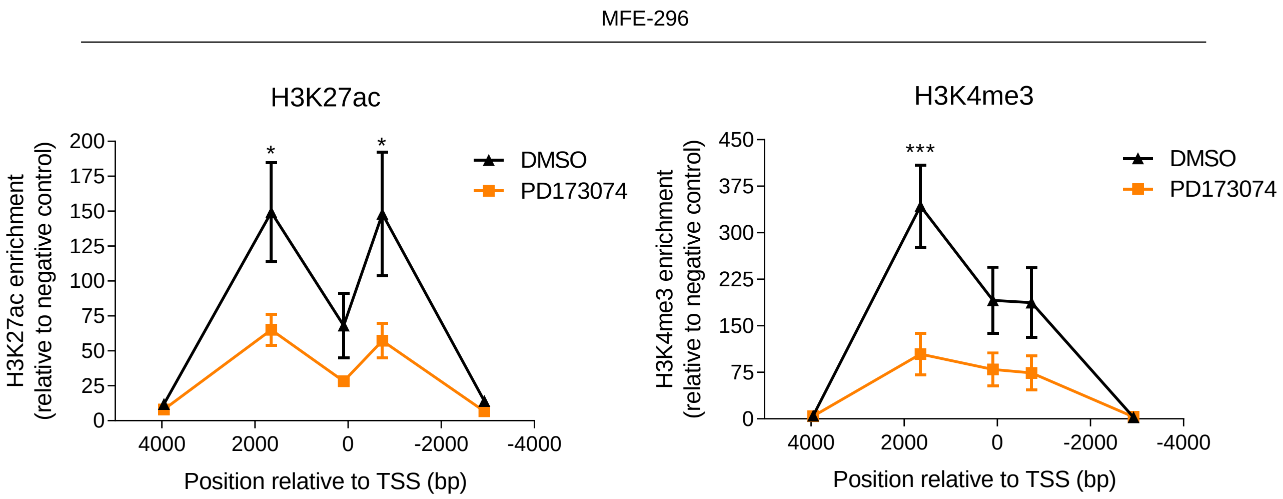

Supplementary Figure S3. MFE-296 cells were treated with 1 μM PD173074 or DMSO control for 7 days followed by ChIP-PCR to quantify H3K27ac (left) and H3K4me3 (right) at the *PHLDA1* locus relative to negative control primers. Data are presented as mean ± SEM of three independent experiments. * P<0.05, *** P<0.001. Two-way ANOVA with multiple comparison test.

Supplementary Figure S4

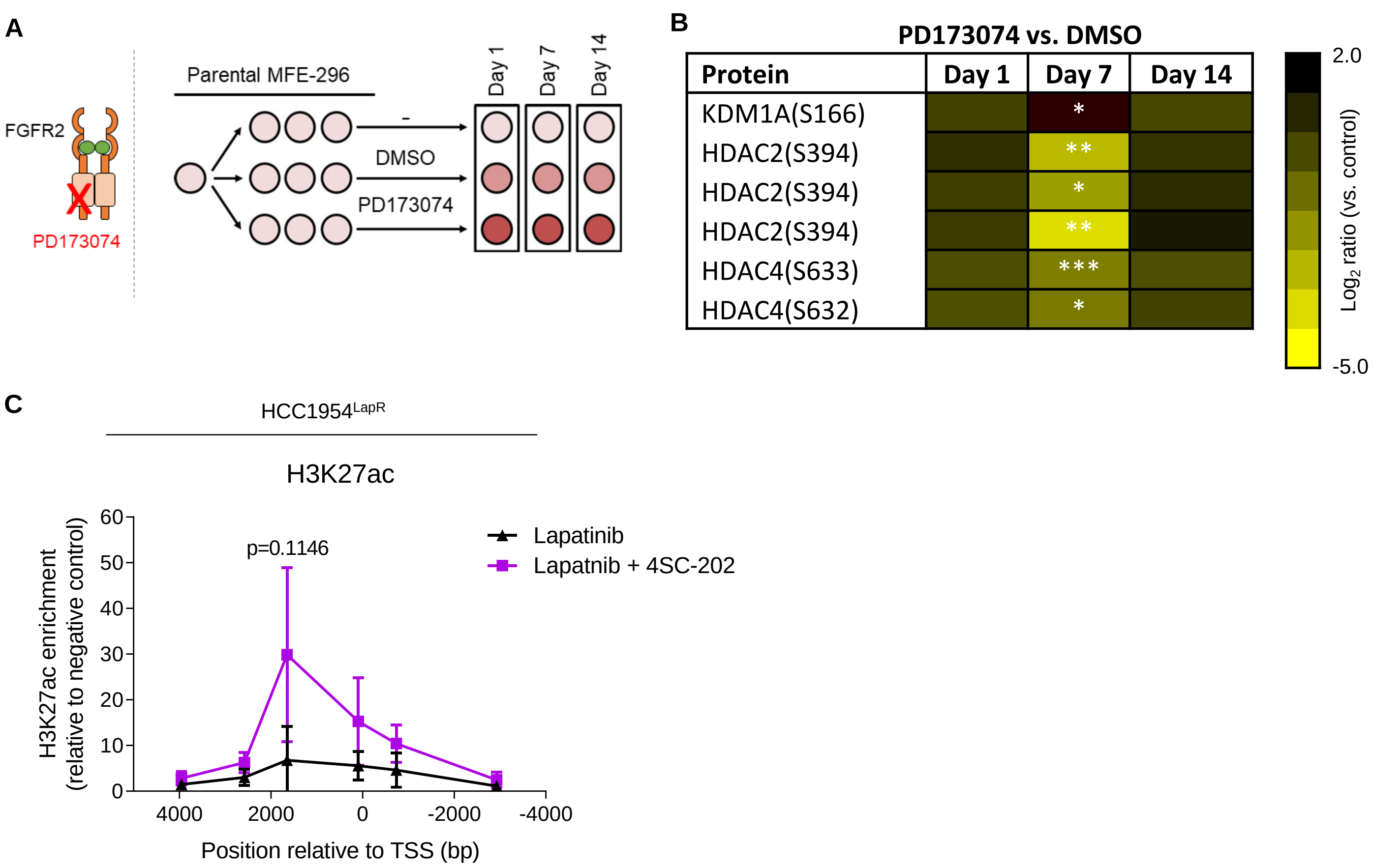

Supplementary Figure S4. **A.** Schematic of phosphoproteomic analysis to discern mechanisms of resistance. MFE-296 cells were treated with 1 µM PD173074 or DMSO control over 1, 7 and 14 days. **B**. Heatmap of epigenetic modifier proteins showing differential phosphorylation following treatment with 1 µM PD173074. * p <0.05, ** P<0.01, *** P<0.001. Two tailed T-Test. **C**. ChIP-PCR analysis of H3K27ac deposition at the *PHLDA1* locus in HCC1954^{LapR} cells cultured in 1 µM lapatinib and treated for 48 hours with 1 µM 4SC-202. Data are presented as mean ± SEM of three independent experiments. Two-way ANOVA with multiple comparison test.

Supplementary Figure S5

A

SKBR3

Cell Proliferation (rows: 4SC-202 (nM); columns: Lapatinib (nM))

4SC-202 (nM)	0	39	78	156	313	625	1250	2500	5000
313	0.55	0.24	0.23	0.16	0.14	0.12	0.12	0.12	0.08
156	0.93	0.51	0.37	0.19	0.16	0.16	0.15	0.13	0.11
78	1.00	0.55	0.39	0.21	0.16	0.16	0.14	0.13	0.12
39	1.02	0.56	0.30	0.19	0.16	0.15	0.14	0.12	0.12
20	1.02	0.44	0.26	0.20	0.16	0.14	0.12	0.12	0.10
9.8	1.04	0.47	0.30	0.20	0.17	0.16	0.14	0.14	0.12
4.9	1.05	0.46	0.27	0.20	0.17	0.16	0.13	0.13	0.10
0	1.00	0.66	0.54	0.49	0.51	0.47	0.50	0.37	0.25

Scale: 0 · 0.1 · 0.3 · 0.4 · 0.5 · 0.6 · 0.7 · 0.9 · 1

Excess above Bliss (rows: 4SC-202 (nM); columns: Lapatinib (nM))

4SC-202 (nM)	0	39	78	156	313	625	1250	2500	5000
313	0.0	0.1	0.1	0.1	0.1	0.1	0.2	0.1	0.1
156	0.0	0.1	0.1	0.3	0.3	0.3	0.3	0.2	0.1
78	0.0	0.1	0.1	0.3	0.3	0.3	0.4	0.2	0.1
39	0.0	0.1	0.3	0.3	0.4	0.3	0.4	0.3	0.1
20	0.0	0.2	0.3	0.3	0.4	0.3	0.4	0.3	0.2
9.8	0.0	0.2	0.3	0.3	0.4	0.3	0.4	0.2	0.1
4.9	0.0	0.2	0.3	0.3	0.4	0.3	0.4	0.3	0.2
0	0.0	0.0	0.0	0.0	0.0	0.0	0.0	0.0	0.0

Scale: 0 · 0.1 · 0.3 · 0.4 · 0.5 · 0.6 · 0.7 · 0.9 · 1

B

SKBR3

Lapatinib (nM): 0, 39, 78 — 4SC-202 (nM): 156, 39, 0

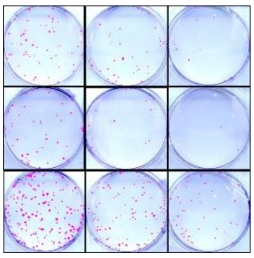

Fold change in colony no. (rows: 4SC-202 (nM); columns: Lapatinib (nM))

4SC-202 (nM)	0	39	78
156	0.33	0.18	0.05
39	0.19	0.06	0.01
0	1.00	0.50	0.29

Scale: 0 · 0.25 · 0.5 · 0.75 · 1

Excess above Bliss (rows: 4SC-202 (nM); columns: Lapatinib (nM))

4SC-202 (nM)	0	39	78
156	0.00	-0.01	0.04
39	0.00	0.00	0.00
0	0.00	0.00	0.00

Scale: 0 · 0.25 · 0.5 · 0.75 · 1

C

SKBR3^{LapR}

Cell Proliferation (rows: 4SC-202 (nM); columns: Lapatinib (nM))

4SC-202 (nM)	0	39	78	156	313	625	1250	2500	5000
313	0.18	0.22	0.19	0.18	0.15	0.15	0.14	0.08	0.06
156	0.38	0.37	0.30	0.38	0.27	0.31	0.27	0.19	0.08
78	0.85	0.62	0.60	0.50	0.52	0.44	0.53	0.20	0.07
39	1.06	0.80	0.70	0.57	0.48	0.44	0.44	0.27	0.06
20	1.08	0.96	0.75	0.63	0.57	0.53	0.54	0.34	0.07
0	1.00	0.66	0.54	0.49	0.51	0.47	0.50	0.37	0.25

Scale: 0 · 0.1 · 0.3 · 0.4 · 0.5 · 0.6 · 0.7 · 0.9 · 1

Excess above Bliss (rows: 4SC-202 (nM); columns: Lapatinib (nM))

4SC-202 (nM)	0	39	78	156	313	625	1250	2500	5000
313	0.0	-0.1	-0.1	-0.1	-0.1	-0.1	-0.1	0.0	0.0
156	0.0	-0.1	-0.1	-0.2	-0.1	-0.1	-0.1	-0.1	0.0
78	0.0	-0.1	-0.1	-0.1	-0.1	0.0	-0.1	0.1	0.1
39	0.0	-0.1	-0.1	-0.1	0.1	0.1	0.1	0.1	0.2
20	0.0	-0.2	-0.2	-0.1	0.0	0.0	0.0	0.1	0.2
0	0.0	0.0	0.0	0.0	0.0	0.0	0.0	0.0	0.0

Scale: 0 · 0.1 · 0.3 · 0.4 · 0.5 · 0.6 · 0.7 · 0.9 · 1

Supplementary Figure S5. A. SKBR3 cells were treated with varying combinations of 4SC-202 and lapatinib for 5 days before cell confluence was measured using an IncuCyte platform. Left panels, cell confluecy fraction relative to vehicle control. Right panels, Bliss Independence analysis of cell proliferation data, where Bliss score >0 represents synergy. Data shown are the mean of three independent experiments. **B**. SKBR3 cells were seeded at low density into 6-well plates and cultured for 21 days in varying combinations of 4SC-202 and lapatinib. Colonies (shown in pink) were visualized by crystal violet (Left panel). Middle panel, normalised colony formation data showing mean fold change. Right panel, Bliss independence analysis. Data shown are the mean of three independent experiments. **C**. SKBR3^{LapR} cells were treated with varying combinations of 4SC-202 and lapatinib for 5 days before cell confluence was measured using an IncuCyte platform. Left panels, cell confluecy fraction relative to vehicle control. Right panels, Bliss Independence analysis of cell proliferation data. Data shown are the mean of three independent experiments.
